# Educating sports people about CPR and first aid in general practice: the Savtember project

**DOI:** 10.1017/S1463423623000282

**Published:** 2023-06-08

**Authors:** Arnaud Maury, Manuel Buet, Emilie Rossignol, Anthony Chapron

**Affiliations:** 1 Université Rennes, CHU Rennes, INSERM, CIC 1414 (Centre d'Investigation Clinique de Rennes), F-35000, Rennes, France; 2 Rennes University, Department of General Practice, Rennes, France

**Keywords:** cardiopulmonary resuscitation, early medical intervention, first aid, general practice, health education, sports

## Abstract

**Aim:**

During an exercise-related sudden cardiac arrest, bystander automated external defibrillator use occurred in a median of 31%. The present study conducted in France evaluated the feasibility and impact of a brief intervention by general practitioners (GPs) to increase awareness about first aid/CPR training among amateur sportspeople.

**Methods:**

In 2018, 49 French GPs proposed a brief intervention to all patients who attended a consultation in order to obtain a medical certificate attesting their fitness to participate in sports. The brief intervention included two questions (Have you been trained in first aid? Would you like to attend a first aid course?) and a flyer on first aid. The GPs’ opinion of the feasibility of the brief intervention was evaluated during a subsequent interview (primary objective). The percentage of sportspeople who started a first aid/CPR course within three months was used as a measure of the effectiveness of the brief intervention (secondary objective).

**Findings:**

Among 929 sportspeople, 37% were interested in first aid training and received the flyer (4% of these started a training course within three months of the brief intervention, a training rate that was 10 times greater than among the general French population), 56% were already trained, and 7% were not interested. All GPs found the brief intervention feasible and fast (<3 min for 80% of GPs). We conclude the brief intervention to promote first aid/CPR awareness is easy to use and may be an effective although limited means of promoting CPR training. It opens a previously unexplored avenue for GP involvement in promoting training.

## Introduction

During an exercise-related sudden cardiac arrest, bystander automated external defibrillator (AED) use occurred in a median of 31% (Grubic *et al.*, 2021). Hence, increasing knowledge of and training in basic life support (BLS) and the use of defibrillators among the lay public would be expected to improve the survival and prognosis of out-of-hospital cardiac arrest (Ro *et al.*, 2019; Vancini *et al.*, 2019; Yu *et al*., 2020). The recent publication of the European Resuscitation Council Guidelines 2021: Systems Saving Lives (Semeraro *et al.*, 2021) provided a comprehensive review of evidence and highlighted the role of lay resuscitation as the most important factor in achieving the best quality outcome of sudden cardiac arrest and made the key recommendation to train as many citizens as possible. This goal has long been recognised. However, its achievement in practice remains problematic and multiple innovative approaches will be essential. Among the extensive literature on means of resuscitation and training, a role for the general practitioner (GP) in promoting training among their patients does not appear to have been considered, beyond the issue of GPs maintaining their own competency (Hollis and Gillespie, 2000). The present study reports on a novel approach by which GPs could increase BLS training rates among a relevant adult population.

The rate of trained individuals among the general population varies across regions in France from 7% to 37% (Karam *et al.*, 2017). First aid/CPR training courses are not compulsory and are usually delivered to volunteers at their workplace or via first aid associations, such as the Red Cross. About 400 000 training certificates are awarded every year (ie, approximately 6 per 1000 inhabitants per year). Thus, targeting sportspeople to increase awareness of the importance of first aid/CPR is proposed as an effective approach, and lack of trained bystanders is one of the main obstacles to performing CPR following an exercise-related cardiac arrest (Matsuyama *et al.*, 2020). In France, recreational sportspeople must see a GP every 1–3 years to receive a sports medical certificate that attests to their fitness to participate in sports training/events. This consultation is part of the GPs’ prevention and screening missions, and we rationalised that it presents a unique opportunity via which to test the feasibility of involving GPs in raising awareness and uptake of BLS training. Patient-centred brief interventions are a well-known and validated prevention tool in primary care, for example targeting health habits (tobacco and alcohol consumption) (Kaner *et al.*, 2018; Wéry *et al.,* 2011), and are compatible with a short GP consultation (Bucher *et al.*, 2017). To our knowledge, no study using a brief intervention to raise awareness about CPR /first aid has been previously published. One study assessed the effectiveness of messages provided in pamphlet form, in increasing the general population’s motivation to learn CPR but it did not involve GPs (McDonald *et al.*, 2010).

Existing public awareness-raising campaigns have been linked to month of the year, such as Stoptober (quitting smoking) and Movember (prostate cancer) (Brown *et al.*, 2014; Thomsen *et al.*, 2016). Although the value of these campaigns is still to be evaluated (Vernon *et al.*, 2021), linking a CPR awareness campaign to September, the month when most sporting activities in Europe start may be effective. Therefore, we tested whether a brief intervention could be used to increase awareness of first aid/CPR training without casting doubt on the benefits of physical activity. Some unforeseen obstacles to this brief intervention could appear (lack of time, lack of information, unease talking about death during sports activity, …), and therefore, we decided to set a feasibility study. In keeping with Stoptober and Movember campaigns, this brief intervention was named ‘Savtember: the saving month’.

The primary objective of the study was to evaluate the feasibility of Savtember intervention to increase awareness of the importance of first aid/CPR knowledge among recreational sportspeople, in general practice. The secondary objective was to evaluate the impact of Savtember on the targeted population, namely the number of sportspeople who underwent first aid/CPR training in the three months following the campaign.

## Materials and methods

A multicentre, prospective, exploratory study was designed to evaluate the feasibility, in the general practice setting, of a brief intervention to increase awareness of the importance of first aid/CPR training among amateur sportspeople who lacked any first aid training.

### Population

No investigator network pre-existed among GPs; hence, GPs in three French administrative districts were contacted by email in July 2018. The website www.savtember.fr was created for the study. The local Conseil de l’Ordre des Médecins (French Medical Association) sent an email message to all listed GPs calling for expressions of interest in the study. GPs who responded positively were met by the study personnel to have the study explained to them. Hence, GPs self-selected for participation in the study.

In September 2018, participating GPs included all adult patients who attended consultations to obtain a sports medical certificate. Although children, under 18 years of age, are receptive to learning CPR, they were excluded to simplify the study design for the GP. The GP recorded whether (i) the patient completed the brief intervention; (ii) the patient was already trained in first aid/CPR; and (iii) the patient received a flyer containing information about training courses from the GP. In the last case, the patient’s phone number was also collected with his/her agreement. Prior to the consultation, patients were informed of the GP’s participation in the study by a notice posted in the waiting room and the GP provided a full explanation during the consultation.

### Brief intervention

Brief interventions are robust and well-established as a public health modality across healthcare settings and socio-cultural groups (Beyer *et al.*, 2019). A BI (ie, one to two questions followed by a brief discussion of the topic between the patient and GP) is a validated and pertinent educational tool for use in the primary care setting (Hargraves *et al.*, 2017). The brief intervention was organised according to three standard steps: *Screening*, *Brief Intervention* and *Referral to ‘Treatment’* (Hargraves *et al.*, 2017).

The *Screening* step followed that used in the smoking cessation model (Stead *et al.*, 2013) and was tested by two GPs prior to initiation of the study: (a) Have you been trained in first aid/CPR? If the answer was YES, the brief intervention was stopped; and (b) would you like to attend a first aid/CPR course? If the answer was YES, the brief intervention was delivered.

In the *Brief Intervention* step, the GP presented the patient with an information flyer and had a short discussion with them on the ‘Savtember campaign’. GPs relied on their own knowledge for this conversation. The flyer was elaborated following the French National Authority for Health recommendations (‘Elaboration d’un document écrit d’information à l’intention des patients et des usagers du système de santé.’, 2008) and carried the pictograms of the French Cardiology Society. It was approved by the French national associations involved in first aid training (the French Red Cross and the Civil Protection Services). The flyer included: data on sudden death in sportspeople, the first aid message (alert emergency services, commence CPR, defibrillate), the contact number of the principal first aid training association and the cost and duration of the training course.

In the *Referral to Treatment* step, it was left up to the patient to act upon the information presented and to contact the first aid training association identified in the flyer.

At the end of the consultation, the GP classified patients who attended for a sports medical certificate into four groups: (i) already trained; (ii) untrained and uninterested in training; (iii) untrained and interested in training; and (iv) not included in the screening (eg, younger than 18 years of age).

### Intervention assessment: feasibility and impact

One month after conclusion of the intervention, the GPs’ opinion of the feasibility of the intervention was evaluated during an individual face-to-face interview that included three questions: (i) Was the intervention feasible? (ii) Was it easy to administer? (iii) How long did it take? The interviews were all conducted by one study author (MB), for consistency.

Three months after the intervention, all patient participants who provided their phone numbers were contacted by SMS to ask whether they had started a first aid training course.

### Data analysis and ethics

The sports medical certificates of participating patients were collected by the principal investigator (MB). They were de-identified and then analysed to determine age, sex and sport type/intensity. Descriptive data were compiled in Excel^©^ and were presented using numbers and percentages, and mean ± standard deviation (SD). Sports were categorised into three groups: low, moderate and high metabolic equivalent (MET), where basal MET was the metabolic equivalent of the oxygen consumption of a 40-year-old man at rest (MET = 1) (Albert *et al.*, 2000).

To assess the validity of the data, the number of sports certificates issued was compared with the number of days on which GPs received patients applying for a sports medical certificate (inclusion days). The appointment diaries for September of 20% of the participating GPs, chosen randomly, were analysed retrospectively to assess the quality of decisions to include patients, by comparing the number of appointments made to obtain a sports medical certificate with the number of medical certificates issued by each GP. In the study, no included patient was refused a sports participation certificate on medical grounds.

The study database was declared to the relevant personal data protection authority (CNIL).

## Results

For this study, 49 GPs were recruited in three French districts. Their characteristics are described in Table 1.

**Table 1.** Description of the 49 GPs who volunteered to test the brief intervention about first aid/CPR training

General Practitioners (*n* = 49)	
Women (%)	55.1
Age, years (SD)	42.4 (±10.6)
Mean number of consultations per hour (SD)	3.4 (±0.7)
Mean number of consultation days per week (SD)	4 (±0.7)
Student supervisor^*^ (%)	42.9
Previously used the brief intervention for smoking (%)	87.7
Working in a multi-GP practice (%)	85.7
Practising sports (%)	73.5
Type of area	
Rural area (%)	22.4
Rural and urban areas (%)	61.2
Urban area (%)	16.3

* General practice teacher receiving medical students at their practice.

The mean (SD) number of inclusion days per GP was 13.5 (±4) over the month of the campaign. The mean (SD) number of sports medical certificates per GP was 19.5 (±16.4) during the same period.

### Patients

**Figure 1.**
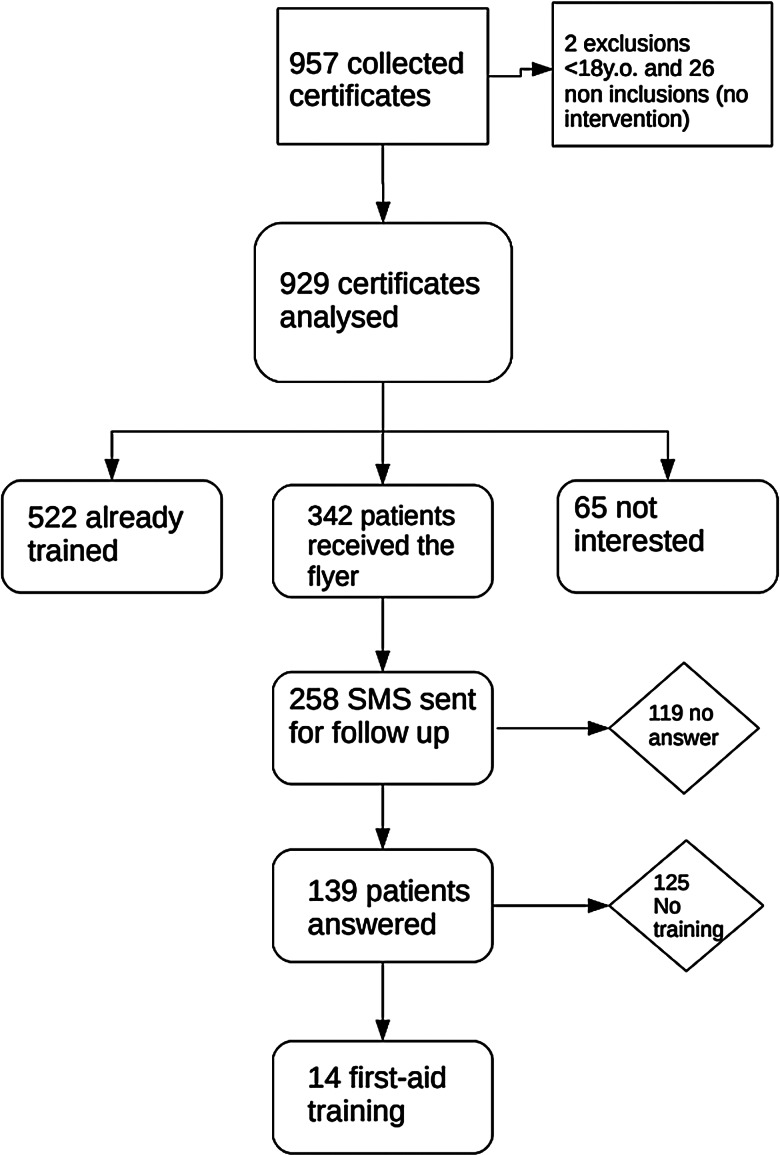
Flowchart describing the 957 collected certificates and 929 patients receiving a brief intervention regarding first aid/CPR training in September 2018 during the Savtember campaign

In total, 957 sports medical certificates were issued by the participating GPs. Analysis of a random sample of the appointment registers of GPs suggested a rate of inclusion of patients in the study that corresponded to 95% of all consultations for a sports medical certificate during the study period. Twenty-six patients were not included (and thus the certificate was not analysed) either because the GP forgot to include them or decided that the patient could not be included. Two certificates were excluded from the analysis because the patients were under the age of 18 years (Figure 1). The mean age of included patients was 43 years (±16) and 60% were women.

In total, data from 929 individual sports medical certificates were analysed. The GPs recorded that 522 (56%) of these patients reported having already been trained in first aid/CPR, 342 (37%) were interested in the topic and received the flyer and brief intervention, and 65 (7%) were not interested (Figure 1). Among the 342 patients who received the flyer, 258 provided their phone number for follow-up.

Among the 955 included sportspeople, 699 practised a high-intensity activity (MET ≥ 6; eg, running, football, gym, badminton), 147 a moderate-intensity activity (MET: 3–5; eg, aquafit, walking and Nordic walking) and 105 a low-intensity activity (MET < 3; eg, yoga, Pilates and slow walking). Activity data for four persons were missing.

### Brief intervention feasibility assessed by GPs

The GPs’ responses to the three questions about the brief intervention indicated that the majority of them found it feasible, easy and fast to administer (Table 2).

**Table 2.** GPs’ opinion of the feasibility and time needed for the brief intervention about first aid/CPR training for sportspeople

General practitioners (*n* = 49)		
The intervention is feasible		49 (100%)
The intervention is easy		48 (98%)
Estimated time needed	Less than 2 min	28 (57%)
	Between 2 and 3 min	11 (23%)
	More than 3 min	10 (20%)

### Intervention impact

Among the 342 patients who received the flyer (Figure 1), 14 (4.1%) reported attending a first aid/CPR training course within three months following the intervention. Their mean age was 41 years, and they all practised an intense sports activity. They represented 1.5% of all included patients. It is noted that 119 of the 342 patients were lost to follow-up, so the proportion might have been higher. Among those who had already been trained, it was not recorded whether any undertook refresher training. Hence, 4.1% may be regarded as a minimum who attended training in first aid/CPR.

## Discussion

With encouragement of fitness and participation in sports, the issue of sudden death in sporting activities may rise, particularly among recreational sportspeople (Vancini *et al.*, 2019). Improved levels of first aid/CPR training are recommended (Semeraro *et al.*, 2021), and a variety of means of promoting it will be needed. The requirement in France of a regularly updated certificate to indicate medical clearance for recreational sport participation, which necessitates a consultation with a GP, provides a potential opportunity to enhance awareness and uptake of first aid/CPR training. Our study tested the feasibility of a brief intervention employed by GPs during these consultations. According to the GPs who participated in this study, the one-month-long brief intervention to promote first aid/CPR training among sportspeople was feasible, quick and easy to do during the consultation.

The secondary aim of the study was to observe rates of uptake of training among the sportspeople studied. Thirty-seven per cent of participants were interested in first aid/CPR training. Three months after the brief intervention, 4.1% of participants who received the brief intervention were known to have attended a first aid training course. All of them practised high-intensity sports, associated with a higher risk of sudden death (Albert *et al.*, 2000).

It was observed that 56% already had first aid/CPR training, a high percentage in the overall French context (Karam *et al.*, 2017) but it accords with the rate of people who had been trained (65%) in the United States in 2017 (Blewer *et al.*, 2017). French labour law mandates the presence of at least one person trained in first aid in the workplace, which may explain this high percentage. However, first aid skills decrease over time. Prompting refresher training may have been a beneficial side effect of our brief intervention campaign, but these data were not captured, so the issue of refresher training would be a useful inclusion in future studies.

### Strengths and weaknesses

It was noted that the GPs who volunteered to participate were younger on average than the overall French GP population (42.4 years *vs* 52 years). It may indicate a recruitment bias. In addition, the proportion of GPs approached who took up the invitation was only 3% of the total pool. Hence, the same level of acceptability of the brief intervention among the broader GP community may not be assumed.

The study also had a declaration bias because GPs were directly asked whether the brief intervention was feasible. The high patient inclusion rate (95%) in itself indicated that the intervention was feasible. The contact with the participating patients after three months was anonymous, to reduce the likelihood of a desirability bias in their response. The analysis of the impact of the intervention should be taken carefully due to the large loss of follow-up. The small sample size may also limit the extrapolation. Further study could include children as they can also learn and perform BLS.

As feasibility among GPs was the primary aim of the study, a single-arm design was appropriately employed. Further study of the secondary aim (uptake of training following brief intervention) would require control groups including non-sportspeople, as well as a non-intervention arm.

### Extrapolating the results to other populations

In most countries, medical sports certificates are not compulsory. Therefore, outside France, employment of a brief intervention such as described herein may have to take place during other consultations with the GP and a choice made either to target only sportspeople or to include all patients. The high observed rate of pre-existing first aid/CPR training among sportspeople in the present study suggests that sportspeople have already a greater interest in this subject.

### The screening and brief intervention tool in general practice

The brief intervention interested approximately one-third of the included sportspeople, but its short-term impact seemed moderate. A longer follow-up (eg, one year) might have captured an increased number of people who started a first aid course. In general, the efficiency of brief interventions is considered moderate and relies on the patient’s motivation to change their behaviour. For example, a review of the effectiveness of minimal interventions for quitting smoking found small effects: an increase of 1–3% in quit rates compared with the non-intervention condition, assuming an unassisted quit rate of 2–3% (Stead *et al.*, 2013). In brief interventions to reduce alcohol consumption, the mean difference after one year was about 8% (Kaner *et al.*, 2018). These rates are comparable to the results of the present study. The seemingly low success rate of our brief intervention may lead to ask whether this is the best intervention type for promoting first aid/CPR training and what should be the GP role. However, the literature does not define standard measure or thresholds of effectiveness for brief interventions. Despite their moderate effectiveness, brief interventions to reduce tobacco and alcohol consumptions are still considered useful. The indirect effect of our CPR training brief intervention on the rate resuscitation by bystanders is harder to measure. Therefore, this new role for the GPs needs to be better investigated before its wider implementation.

The choice of a screening and brief intervention was relevant because it requires minimal or no training for the GP administering it making it low-cost, reproducible and fast. The GPs relied on their own knowledge for the conversation on first aid/CPR training. Although the motivational approach was mainly based on letting patients explore their point of view, the discussion component could be improved by preparing specific messages to be delivered and a personalised follow-up with the patient. Specific messages would also make this intervention easier to replicate.

### Implications for future first aid training campaigns

It is noted that education in BLS, not defibrillator density, was the most important predictor of survival in out-of-hospital cardiac arrest (Karam *et al.*, 2017). In this context, increasing awareness of the importance of first aid training could reasonably be part of the GPs’ mission. It is known that GPs’ own skills in BLS are insufficient (Hollis and Gillespie, 2000), which might limit their willingness to talk about it with their patients. Regular training for emergency situations and a regular public health campaign on the issue may help motivate GPs to enhance their own skills. Financial incentives could also help to develop this culture among GPs.

## Conclusions

This observational study demonstrated the feasibility of a brief intervention by GPs about first aid training/CPR targeted to sportspeople. It also found that one in three sportspeople was interested in talking about first aid training and that over 50% reported already having been trained. To enhance its impact, the intervention could be proposed to all patients, not just to active sportspeople, though sportspeople may have a greater intrinsic interest. Further studies that include a control group without coordinated intervention could measure more precisely its interest from a public health perspective as it remains unclear whether or not this intervention should be done in primary care.

Given the emphasis on BLS training worldwide, the present study provides a unique insight into a means by which GPs could contribute to raising awareness.

## Data Availability

Data supporting the findings of this study are available from the corresponding author [AM] on request.
